# MALAT1 Polymorphisms and Lung Cancer Susceptibility in a Chinese Northeast Han Population

**DOI:** 10.7150/ijms.73026

**Published:** 2022-07-11

**Authors:** Guanghui Tong, Weiwei Tong, Ran He, Zhigang Cui, Sixuan Li, Baosen Zhou, Zhihua Yin

**Affiliations:** 1 Department of Epidemiology, School of Public Health, China Medical University, Shenyang 110122, P.R. China.; 2 Department of Obstetrics and Gynecology, Liaoning Provincial Hospital for women and children, Shayang Street, Heping District, Shenyang 110122, P.R. China.; 3 Clinical Laboratory, Shengjing Hospital of China Medical University, Shenyang, P.R. China.; 4 Department of Clinical Epidemiology and Center of Evidence Based Medicine, The First Hospital of China Medical University, No. 155 Nanjing Bei Street, Heping District, Shenyang 110001, P.R. China.

**Keywords:** Lung cancer, LncRNA, MALAT1, Single nucleotide polymorphism, Interaction.

## Abstract

**Background:** LncRNA MALAT1 (metastasis-associated lung adenocarcinoma transcript 1) was competitive endogenous RNA (ceRNA) involved in various molecular processes for metastasis development in lung cancer. Single nucleotide polymorphisms (SNPs) in MALAT1 gene might be predictive markers for lung cancer. In our study, we selected rs619586 and rs3200401 in MALAT1 gene to explore their effects on lung cancer susceptibility.

**Methods:** The case-control study included 444 lung cancer cases and 460 healthy controls. Genotyping was performed by Taqman allelic discrimination method. Logistic regression, Student *t*-test, and Chi-square test (*χ^2^*) were used to analyze the data.

**Results:** The findings of the study showed that rs3200401 was significantly associated with the risk of non-small cell lung cancer (NSCLC) and lung squamous cell carcinoma (LUSC). Compared with homozygous CC genotype, CT heterozygous genotype decreased risk of NSCLC (*P^a^* = 0.034) and LUSC (*P^a^* = 0.025). In addition, no statistical association was detected between rs619586 and lung cancer susceptibility. The interactions between genes and cigarette smoking were discovered via crossover analysis. However, there were no remarkable gene-environment interactions in additive and multiplicative model.

**Conclusion:** Rs3200401 in lncRNA MALAT1 was associated with the susceptibility of non-small-cell lung cancer and lung squamous cell carcinoma. The gene-environmental (cigarette smoking) interactions were not notable.

## Introduction

In recent years, a very important factor causing human death was malignant tumors. In particular, the incidence and mortality of lung cancer are always at the forefront 1-3. The global data (GLOBOCAN 2018) predicted that the number of cases in Asia accounted for nearly half of all new malignancies (18.1 million) worldwide, and nearly 70% of cancer deaths are Asian 4. Cigarette smoking is one of the environmental risk factors leading to lung cancer. But it is estimated that half of all new cases were non-smokers or smokers who quit many years ago 5. Therefore, smoking cannot fully explain the cause of the disease. Other studies had shown that genetic factors also play a crucial role in the process of lung cancer 6-13.

Non-coding RNA (ncRNAs) are divided into two types by length: small ncRNAs (< 200 bp) and lncRNAs (> 200 bp)^14^. Many studies have increasingly proved that long non-coding RNAs play critical roles in biological process 15-22. Genome-wide association studies (GWAS) have showed that numerous single nucleotide polymorphisms (SNPs) are associated with some diseases.

LncRNA MALAT1 located at 11q13, which is also known as NEAT2 (nuclear-enriched abundant transcript 2). MALAT-1 was first discovered in a study of non-small cell lung cancer 23. Since the discovery of MALAT1, it had played an important role in the occurrence, development, metastasis, drug resistance and clinical treatment of the disease 16, 24-30, especially in lung cancer. Moreover, many papers had reported the risk of MALAT1 polymorphisms and diseases susceptibility 31-44. However, few studies have investigated about MALAT1 polymorphisms and the risk of lung cancer. Therefore, the study recruited Chinese Northeast Han Population as the research objects and performed a case-control study to explore the association between MALAT1 polymorphisms and susceptibility of lung cancer.

## Material and Methods

### Study subjects and data collection

The Institutional Review Board of the China Medical University approved the case-control study which was carrying out in Shenyang, Liaoning Province. All the subjects were from the Chinese Northeast Han Population. Cases were from several hospitals in Shenyang. Inclusion and exclusion criteria of the cases were mentioned in our previous studies 39, 45-47. And the healthy controls were from medical examination centers of hospital. Both cases and controls who smoke more than 100 cigarettes a lifetime were defined as smokers. After signing the informed consent form and filling out the personal information questionnaire, all participants were collected 5mL of venous blood and stored in the -20 ℃ environment for the next step of DNA extraction.

### SNPs selection and Genotyping

The method of screening two SNPs was the same as the previous research 48. The minimum allele frequencies (MAF) of the two SNPs (rs619586, rs3200401) were more than 0.05 in Chinese Han population (CHB). Genotyping were conducted by a Real-Time Polymerase Chain Reaction (PCR) with the TaqMan assay. 10% of the samples were randomly selected for repeated experiments, and the genotyping results were consistent.

### Statistical analysis

The data were performed by *SPSS* software 20.0 (IBM SPSS, Inc., Chicago, IL, USA). The distributions among differences with cigarette smoking, age and gender were assessed by Chi-square test (*χ^2^*) and student *t*-test. Goodness-of-fit* χ^2^* was used to compute the value of Hardy-Weinberg equilibrium (HWE) in the control group. Logistic regression analysis was performed to analysis the experimental data (Indicators: odds ratios (ORs) and 95% confidence intervals (CIs)). And the two indicators were adjusted by gender, age, and cigarette smoking. The interactions were described by additive and multiplicative models. *P-*value <0.05 (two-sided) was defined as statistically significant.

## Results

### Subject characteristics

The study included 460 healthy controls and 444 lung cancer cases with the average ages of 58.03 and 59.45, respectively. There were no significant differences in gender and age between case and control group, while there was a statistical difference in cigarette smoking (*P*<0.001). Case group contained 213 lung adenocarcinoma (LUAD) cases, 145 lung squamous cell carcinomas (LUSC) cases and 86 small cell lung cancers (SCLC). The detailed information was shown in Table 1. In addition, the values of HWE were more than 0.05(*P* = 0.543 for 619586, *P* = 0.061 for rs3200401).

### Genotype distribution and Lung Cancer susceptibility

The association of the two SNPs with lung cancer, NSCLC, LUAD and LUSC were shown in Table 2 and Table 3. The results demonstrated that there was no significant difference between the rs619586 and the risk of the lung cancer and other pathological classifications. However, we found that the relationship between rs3200401 and non-small cell lung cancer risk (CT vs. CC: OR^a^ = 0.707, 95%CI = 0.513-0.974, *P* = 0.034). At the same time, rs3200401 and lung squamous cell carcinoma had statistical differences in the two gene models (CT vs. CC: OR^a^ = 0.581, 95%CI = 0.361-0.935, *P* = 0.025; TT+CT vs. CC: OR^a^ = 0.634, 95%CI = 0.403-0.997, *P* = 0.048).

### Interactions between SNPs and Cigarette Smoking

In Table 4, crossover analysis was performed to estimate the interaction between the two SNPs and cigarette smoking in lung cancer. The combined effect of the dangerous genes and cigarette smoking increased the risk of disease to a certain extent. The results showed that the interaction between two SNPs and cigarette smoking were found in lung cancer and NSCLC. Table 5 presented that rs619586 and rs3200401 had no additive interaction with cigarette smoking exposure. Similarly, there were no interactions between the two SNPs and cigarette smoking exposure in multiplicative model (Table 6).

## Discussion

LncRNA MALAT1 was first reported in non-small cell lung cancer 23, but few studies have published on its polymorphisms and susceptibility to lung cancer. In view of the current high mortality and morbidity of lung cancer, many mechanism studies also had shown that MALAT1 was related to the occurrence, development and prognosis of lung cancer. Therefore, it was necessary to study its genetic polymorphisms and susceptibility to lung cancer. In our study, we selected rs619586 and rs3200401 to explore their risk with lung cancer susceptibility.

**Figure 1:**
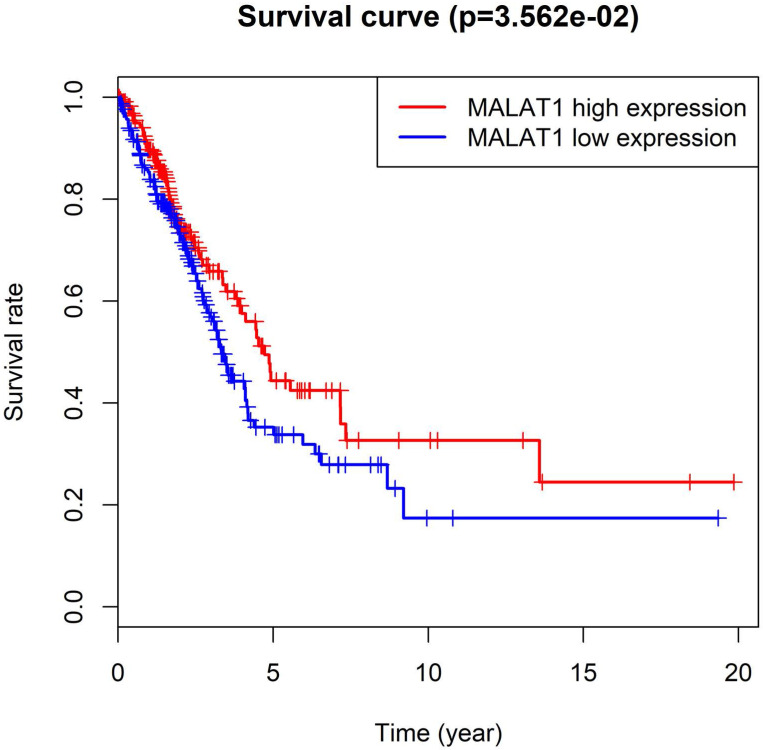
Malate1 expression and survival rate

MALAT1, also known as NEAT 2, was competitive endogenous RNA (ceRNA) involved in various molecular processes 49. Particularly, MALAT1 was a predictive marker for metastasis development in lung cancer 13. A study discovered lncRNA-MALAT1 participates in NSCLC progression by targeting miR-202 8. Furthermore, increased levels of lncRNA MALAT1 could promote brain metastasis of lung cancer by inducing EMT 7. High expression of MALAT1 promoted the progression of non-small cell lung cancer through the ERK / MAPK signaling pathway 6. Interestingly, the results of the Cancer Genome Atlas (TCGA) analysis showed that the high expression of MALAT1 in lung adenocarcinoma has a higher survival rate than the low expression in Figure 1 (54 normal / 497 LUAD tissues). These differences required a series of experiments to verify.

Some studies have shown that lncRNA SNPs are associated with cancer risk and are potential predictive biomarkers of cancer risk 30. The association between rs3200401 polymorphism of MALAT1 in terms of drug efficacy and toxicity has been confirmed, and the rs3200401 CT genotype can be used as a toxicity biomarker 50. The association between rs3200401 polymorphism of MALAT1 in terms of drug efficacy and toxicity has been confirmed, and the rs3200401 CT genotype can be used as a toxicity biomarker 30. It can be seen that rs3200401 C and T alleles can change the structural characteristics of MALAT1 and reshape the expression level of cancer-related genes, thus participating in the occurrence and development of cancer 49, 51. There is an association between MALAT1 rs3200401 and lymph node status, Perhaps further research can reveal their potential as genetic biomarkers for Colorectal cancer (CRC) 51. Wang et al. showed that T allele of rs3200401 was protective factor in survival outcomes of patients with advanced lung adenoma^9^. In a recent meta-analysis that did not include lung cancer, the researchers did not find a relationship between the MALAT1 rs3200401 polymorphism and overall cancer risk, rs3200401 C > T polymorphism plays different roles in cancers 52. Our study revealed that rs3200401 reduces the risk of non-small cell lung cancer and lung squamous cell carcinoma. Similar to our results, Ding et al. found that the rs3200401 CT and TT genotypes significantly reduced the risk of developing Oral squamous cell carcinoma (OSCC) 53. However, MALAT1 rs3200401 was significantly associated with increased disease risk in esophageal squamous cell carcinoma (ESCC), CRC and atrophic gastritis 32, 51, 54. Moreover, the polymorphisms of rs3200401 were not statistically significant in many diseases, such as breast cancer (BC), multiple sclerosis (MS), ischemic stroke (IS) and coronary artery disease (CAD) and so on^33^, ^35^-^37^, ^39^, ^42^, ^44^. MALAT1 rs619586 G allele enhanced the binding of mir-214 to MALAT1 and promoted OSCC development 53. In breast cancer (BC), Peng et al. found rs619586 AG genotype had a lower risk of BC 39. Furthermore, patients with the G allele of the rs619586 polymorphism had a significantly increased risk of developing a high-grade Gleason grade in prostate cancer 55. MALAT1 rs619586 A>G has A protective effect on meningioma invasion by inhibiting the activation of collagen type V alpha (COL5A1) downstream gene 56. A previous study showed that MALAT1 rs619586 polymorphism significantly reduced the risk of lung cancer 57. But in this study, rs619586 and lung cancer were not statistically significant difference, the differences were found in many other diseases 33, 35, 37-39, 44. We did not find gene-environment interactions in multiplication and addition models. The reason might be that the sample size is not large enough.

To obtain more accurate results, OR and 95% CI were adjusted by age, gender, and cigarette smoking. At the same time, we strictly control the standards of each step. However, this study also had some inadequacies with all similar articles. Factors such as selection bias, sample size, and statistical power may affect the results of our study. Functional mechanism experiments would be needed to verify this result in the future.

## Conclusion

The polymorphisms rs3200401 in MALAT1 was associated with the risk of non-small cell lung cancer and lung squamous cell carcinoma in Chinese Northeast Han population. The gene-environmental (cigarette smoking) interactions were not notable.

## Figures and Tables

**Table 1:** Distribution of demographic variables in lung cancer and controls.

Risk factor	Lung cancer (%)	Control (%)	*P*
	(n=444)	(n=460)	
Age(mean ± SD)	59.45±10.768	58.03±13.995	0.087
≤60	226(50.9)	227(49.3)	0.641
>60	218(49.1)	233(50.7)	
Gender			0.530
Male	263 (59.)	263(57.2)	
Female	181 (40.8)	197(42.8)	
Smoking status			<0.001
Ever	227(51.1)	161 (35.0)	
Never	217(48.9)	299(65.0)	
Pathological type			
LUAD	213(48.0)		
LUSC	145(32.7)		
SCLC	86(19.4)		

LUAD: lung adenocarcinoma, LUSC: lung squamous cell carcinoma, SCLC: small cell lung cancer.

**Table 2:** The association of the two SNPs with lung cancer and non-small cell lung cancer risk.

Genotyping	Control (%)	Lung cancer			Non-small cell lung cancer		
	(n=460)	Cases(%)(n=444)	ORª(95%CI)	*Pª* value	Cases (%)(n=358)	ORª(95%CI)	*Pª* value
rs619586							
AA	372(80.9)	360(81.1)	1.00(Ref)		288(80.4)	1.00(Ref)	
GA	82(17.8)	82(18.5)	1.057(0.749-1.493)	0.751	68(19.0)	1.115(0.776-1.604)	0.555
GG	6(1.3)	2(0.5)	0.409(0.080-2.097)	0.284	2(0.6)	0.486(0.095-2.489)	0.387
GG+GA vs. AA			1.016(0.724-1.424)	0.929		1.074(0.752-1.532)	0.696
GG vs. AA+GA			0.406(0.079-2.076)	0.279		0.478(0.093-2.442)	0.375
A allele	826(89.8)	802(90.3)	1.00(Ref)		644(89.9)	1.00(Ref)	
G allele	94(10.2)	86(9.7)	0.942(0.692-1.282)	0.705	72(10.1)	0.982(0.711-1.358)	0.915
rs3200401							
CC	309(67.2)	321(72.3)	1.00(Ref)		263(73.5)	1.00(Ref)	
CT	143(31.1)	112(25.2)	0.755(0.560-1.017)	0.065	85(23.7)	0.707(0.513-0.974)	0.034
TT	8(1.7)	11(2.5)	1.264(0.494-3.232)	0.625	10(2.8)	1.345(0.516-3.508)	0.545
TT+CT vs. CC			0.783(0.585-1.047)	0.099		0.743(0.545-1.014)	0.061
TT vs. CC+CT			1.368(0.537-3.487)	0.511		1.479(0.569-3.846)	0.422
C allele	761(82.7)	754(84.9)	1.00(Ref)		611(85.3)	1.00(Ref)	
T allele	159((17.3)	134(15.1)	0.851(0.662-1.093)	0.206	105(14.7)	0.822(0.629-1.076)	0.153

^a^ Adjusted by age, gender, smoking. OR, odds ratio; CI, confidence interval.

**Table 3:** The association of the two SNPs with lung adenocarcinoma and lung squamous cell carcinoma risk.

Genotyping	Control (%)	Lung adenocarcinoma			Lung squamous cell carcinoma		
	(n=460)	Cases (%)(n=213)	ORª(95%CI)	*Pª* value	Cases (%)(n=145)	ORª(95%CI)	*Pª* value
rs619586							
AA	372(80.9)	171(80.3)	1.00(Ref)		117(80.7)	1.00(Ref)	
GA	82(17.8)	40(18.8)	1.042(0.681-1.596)	0.848	28(19.3)	1.244(0.747-2.072)	0.401
GG	6(1.3)	2(0.9)	0.913(0.179-4.671)	0.913	0(0)	-	-
GG+GA vs. AA			1.035(0.683-1.569)	0.872		1.144(0.691-1.894)	0.600
GG vs. AA+GA			0.907(0.178-4.634)	0.907		-	-
A allele	826(89.8)	382(89.7)	1.00(Ref)		262(90.3)	1.00(Ref)	
G allele	94(10.2)	44(10.3)	1.012(0.694-1.477)	0.950	28(9.7)	0.939(0.602-1.464)	0.782
rs3200401							
CC	309(67.2)	153(71.8)	1.00(Ref)		110(75.9)	1.00(Ref)	
CT	143(31.1)	55(25.8)	0.796(0.549-1.154)	0.229	30(20.7)	0.581(0.361-0.935)	0.025
TT	8(1.7)	5(2.3)	1.288(0.410-4.044)	0.665	5(3.4)	1.486(0.422-5.234)	0.538
TT+CT vs. CC			0.822(0.573-1.180)	0.289		0.634(0.403-0.997)	0.048
TT vs. CC+CT			1.377(0.441-4.304)	0.582		1.706(0.486-5.988)	0.404
C allele	761(82.7)	361(84.7)	1.00(Ref)		250(86.2)	1.00(Ref)	
T allele	159((17.3)	65(15.3)	0.862(0.629-1.180)	0.354	40(13.8)	0.766(0.526-1.114)	0.162

^a^ Adjusted by age, gender, smoking. OR, odds ratio; CI, confidence interval.

**Table 4:** Crossover Analysis of Interaction Between the two SNPs and cigarette smoking with lung Cancer and non-small Cell lung cancer risk.

	Control (%)	Smoking	Lung Cancer			Non-small cell lung cancer		
	(n=460)		Cases(%)(n=444)	OR(95%CI)	*P* value	Cases(%)(n=358)	OR(95%CI)	*P* value
**rs619586**								
AA	235(51.1)	Never	177(39.9)	1.00(ref)		148(41.3)	1.00(ref)	
GA+GG	64(13.9)	Never	40(9.0)	0.830(0.534-1.289)	0.407	34(9.5)	0.844(0.530-1.342)	0.472
AA	137(29.8)	Ever	183(41.2)	1.773(1.320-2.382)	0.000*	140(39.1)	1.623(1.187-2.218)	0.002*
GA+GG	24(5.2)	Ever	44(9.9)	2.434(1.427-4.153)	0.001*	36(10.1)	2.382(1.366-4.153)	0.002*
**rs3200401**								
TT+CT	94(20.4)	Never	62(14.0)	1.00(ref)		48(13.4)	1.00(ref)	
CC	205(44.6)	Never	155(34.9)	1.146(0.782-1.681)	0.484	134(37.4)	1.280(0.849-1.929)	0.238
TT+CT	57(12.4)	Ever	61(13.7)	1.623(1.001-2.630)	0.049*	47(13.1)	1.615(0.960-2.715)	0.071
CC	104(22.6)	Ever	166(37.4)	2.420(1.616-3.623)	0.000*	129(36.0)	2.429(1.575-3.746)	0.000*

^a^ adjusted by age and gender. OR, odds ratio; CI, confidence interval, *Indicates statistical significance (P<0.05).

**Table 5:** Addictive interaction measures between the two SNPs and cigarette smoking exposure.

		Lung cancer			Non-small cell lung cancer	
SNP	Measure	Estimate	95%CI	Measure	Estimate	95%CI
rs619586	RERI	0.831	-0.484, 2.146	RERI	0.916	-0.420, 2.251
	AP	0.341	-0.056, 0.739	AP	0.384	-0.011, 0.779
	S	2.377	0.647, 8.731	S	2.964	0.597, 14.726
rs3200401	RERI	0.651	-0.223, 1.526	RERI	0.534	-0.407, 1.476
	AP	0.269	-0.075, 0.613	AP	0.220	-0.154, 0.594
	S	1.847	0.611, 5.578	S	1.597	0.570, 4.472

RERI, relative excess risk due to interaction; AP, attributable proportion due to interaction; S, synergy Index; 95 % CI, 95% confidence interval.

**Table 6:** Multiplicative interaction between the two SNPs and cigarette smoking exposure

		Lung Cancer			Non-small cell lung cancer	
SNPs	Variables	OR^a^ (95% CI)	*P^a^* value		OR^a^ (95% CI)	*P^a^* value
rs619586	GA+GG vs. AA	0.829(0.533-1.291)	0.407		0.856(0.536-1.366)	0.514
	Smoking	2.055(1.468-2.877)	0.000		1.837(1.286-2.623)	0.001
	Interaction	1.660(0.822-3.350)	0.157		1.758(0.842-3.672)	0.133
rs3200401	CC vs. CT+TT	1.142(0.777-1.679)	0.499		1.261(0.834-1.908)	0.272
	Smoking	1.892(1.130-3.169)	0.015		1.853(1.063-3.230)	0.030
	Interaction	1.291(0.721-2.312)	0.390		1.158(0.621-2.160)	0.644

^a^ adjusted by age, gender. CI confidence interval, OR odds ratio.

## Abbreviations

BC: breast cancer
CAD: coronary artery disease
COL5A1: collagen type V alpha
CHB: Chinese Han population
Cis: confidence intervals
ceRNA: competitive endogenous RNA
ESCC: esophageal squamous cell carcinoma
GWAS: Genome-wide association studies
HWE: Hardy-Weinberg equilibrium
IS: ischemic stroke
LUAD: lung adenocarcinoma
LUSC: lung squamous cell carcinoma
MALAT1: metastasis-associated lung adenocarcinoma transcript 1
MAF: minimum allele frequencies
MS: multiple sclerosis
NcRNAs: Non coding RNA
NSCLC: non-small cell lung cancer
NEAT2: nuclear-enriched abundant transcript 2
ORs: odds ratios
OSCC: Oral squamous cell carcinoma
PCR: polymerase chain reaction
SNPs: single nucleotide polymorphisms
SCLC: small cell lung cancers
